# Scrofula; Its Nature, Its Causes, Its Prevalence, and the Principles of Treatment

**Published:** 1846-07

**Authors:** 


SCROFULA; ITS NATURE, ITS CAUSES, ITS PREVALENCE, AND THE PRINCIPLES OF TREATMENT. By *Benjamin Phillips*, F.R.S. Assistant Surgeon to the Westminster Hospital. 8vo. pp. 397. London. Bailliere, 1846.

THIS work reflects very great credit on Mr. Phillips as a most ardent and indefatigable enquirer after truth. The accumulation of so many details must have cost him a vast deal of patient research, and their arrangement much tedious and elaborate study; for his investigations have extended not merely to the public institutions within his reach, and to those at home, but to very many on the Continent of Europe, and even to some in America and in the East Indies. By putting himself in communication with medical men in different parts and regions of the world, and thus collecting information from a variety of sources, he reasonably hoped that he might be enabled to delineate, more accurately than preceding enquirers had been able to do, the natural history of the disease he had undertaken to illustrate, searching out its causes, and shewing what amount of influence such agencies as climate, food, occupation, mode of life, &c. have upon its development, and thus to suggest a more successful plan for its treatment, preventive as well as curative. One of the chief difficulties, attending all such wide-spread enquiries, arises almost necessarily from the circumstance that medical men are by no means agreed among themselves as to the essential characters of the *object* or *condition* to which the appellation of Scrofula is to be given. What are we exactly to mean by this term? What are the distinctive features of the disease? and how are we to recognise its existence? Unless all enquirers are agreed upon these points, it is quite obvious that we can never reasonably expect to arrive at any thing like satisfactory general conclusions, when the data and materials, from which these conclusions are to be drawn, have been obtained from different sources. Mr. Phillips was fully aware of this difficulty *in limine*, and accordingly one of his first objects was to fix upon certain visible and readily discoverable features or phenomena, the presence of which was to be regarded as positive and trustworthy evidences of the existence of Scrofula. Let us now see what these features are said to be.

At page 9, he "defines the ordinary marks of Scrofula to mean enlarged glands discoverable by the touch or the sight, sinuses or ulcerations resulting from them, and ordinary scrofulous bones or joints." At page 26, it is said that " Scrofula is a disease of the constitution, and that it is most clearly manifested by certain external signs, of which swelling of the subcutaneous lymphatic ganglia is the most conclusive." A little farther on, it is defined to be " a disease, manifested by a peculiar deposit in the subcutaneous lymphatic glands;" the mere enlargement of the glands, although affording a strong presumption, being not regarded *per se* as an absolute proof, that the constitution of the individual is scrofulous; for our author expressly says that, " unless the swelling of the gland be accompanied by a deposit of a product, hereafter to be described, known as *scrofulous matter*, the proof of a scrofulous constitution is, in my judgement, wanting." Still more emphatic is the language used in the very next page, where Mr. P. expresses his belief that " diseases regarded as scrofulous, but in which no scrofulous matter is present, are not scrofulous at all, but simply the result of such low inflammatory action as is often set up in a debilitated state of the constitution." In following out his views, we shall find that our author comes to the conclusion that " the accumulation of certain morbid materials in the blood constitutes what is known as the scrofulous diathesis or constitution, and their deposition in the subcutaneous lymphatic glands constitutes what we know as scrofula." Many readers will doubtless at once object to so much stress being laid upon the change in the *subcutaneous glands*, as if the mere fact of their enlargement constituted the pathognomonic sign or evidence of the existence of the disease. Mr. Phillips himself seems to afford more than one argument against this doctrine; for we find him admitting, in one passage, that " the bronchial glands are affected (*with scrofulous deposit,—Rev.*) more than twice as often as those of the mesentery, the latter four times as often as those of the neck, though in many respects less exposed, and the last-named glands four times as those of the axilla and groins." If such then be the case, does not this very admission shew how fallacious it may be to take an enlarged state of the cervical glands as a safe or trustworthy test to discover the actual or relative frequency of scrofulous disease among any number of individuals? We must confess that, throughout the entire perusal of Mr. Phillips' most laborious and praiseworthy investigations, this objection has continually recurred to our mind. His correspondent, Dr. Griffin of Limerick, seems to take the same view; for we find him saying:

" With regard to enlarged glands, whether visible or perceptible to the touch, I doubt their being decided indications of a scrofulous tendency; eruptions on the skin, and especially on the head, will produce them, and such affections are extremely common in Ireland. Most of the enlargements included in the above Tables were of a very trifling nature; indeed the vast majority of them were so, (about 80 per cent. or thereabouts). But I made it a rule (as you seemed to require a test), to include all such cases as were distinctly though very slightly visible, as well as all such as were distinctly, though often but slightly, perceptible to the touch." P. 318.

That it is far from being easy, even in our author's opinion, to determine the actual existence of Scrofula in an individual, is abundantly obvious from the following passages:—

" In a constitution favourable for the deposit of scrofulous matter, I believe there are no features, in the absence of the tumor, so constant and so conclusive as to justify a reliance upon them, in pronouncing an opinion whether a constitution be scrofulous or not. It is certain that the ordinary tests are fallacious; I know that the major part of them may be observed, again and again, without any other evidence that the constitution is tainted with Scrofula. We may even have enlarged glands, while no product such as that which I have alluded to, is deposited; although, in the absence of any source of irritation, enlarged subcutaneous glands constitute grounds for grave suspicion that the constitution is scrofulous. Thus, whatever may be the constitutional peculiarity, however marked may be the general physiognomy by what is called the scrofulous diathesis, we have no certain sign of the existence of the disease until sufficient evidence can be obtained that the deposit has taken place. The constitution may suffer long before such a deposit is made, and the glands themselves may be swelled without presenting in their substance a scrofulous deposit; indeed the deterioration of the system proceeds so slowly, that although the tendency be directly onwards from the period when the gland is simply enlarged, to that when the deposit would ordinarily occur—in that interval favourable or unfavourable circumstances may be experienced, and no deposit may take place;—on the one hand, the constitution may improve and the glandular swelling may subside; on the other, the ailing child's life may be cut short by other diseases before the proof of Scrofula is complete." P. 33.

Again, a few pages farther on, Mr. P. remarks that

"The condition of the economy, then, which is favourable to the formation of scrofulous matter is not Scrofula, but a diathesis, or disposition; it may exist long, it may cause the tumefaction of many glands, but, under favourable circumstances, it may disappear without the deposit of a single particle of that product whose presence in the glandular structure is, I conceive, necessary to constitute Scrofula. Again, I say, the condition of the system favourable to this deposit is marked by *no certain* external signs, up to the moment when the glands become tumid. The child may be fair or dark, pallid or ruddy, well fed, clad, and lodged, or all these may be the worst possible; he may be the child of wealth or of poverty; he may live in town or country; he may be the child of old or young, of healthy or sickly parents; he may be born and live within the tropics, or in the arctic circle; under all these circumstances, as we shall see hereafter, Scrofula may be developed." P. 37.

The Physical Characters of "the deposit whose presence in the glandular structures is (supposed to be) necessary to constitute Scrofula," are thus described:—"To the naked eye, it is presented in the form of an amorphous greyish, buffish, or yellowish mass, irregularly granular, and not unlike moist old cheese."

The Microscopical Characters of this morbid product have been examined by Professor Albers of Bonn, and other continental pathologists, as well as by our countrymen, Messrs. Dalrymple and Gulliver. Mr. D. has communicated the following description to our author:—

"The whole material is composed of disintegrated tissue, granular molecules, irregular exudation corpuscles, and in which the nucleolus is seldom to be recognised, and a considerable quantity of oil globules, which may be abstracted by boiling in æther, and recovered by evaporation on a plate of glass.

"In acute or chronic inflammation of the glands, in otherwise healthy subjects, in whom no particular morbid disposition exists, the exudation corpuscles, by what appears to be a law of vitality, proceeds to the development of a cyst around the nucleus, or cyto-blast; and this nucleus even splits into two or more, and hence a pus globule is formed. At this point, however, the process stops, and the pus globule subsequently disintegrates, and is resolved into granular and fluid matter. During the development of the cell and fissure of the nucleus, a pus globule may be said to be an organic and vitalised body, deriving its means of increase from the blastema around.

"The exudation corpuscle, however, is capable of a much higher degree of organization, and under favourable circumstances, the cell germ produces its cell, the cell elongates, and either fibre or filament is produced, as in the healing of a wound.

"In this scrofulous matter it appears that the exudation corpuscles do not possess even that feeble vital power, which induces the further change into pus, and therefore it passes from the nucleolated cyto-blast into an irregular granular body, (disintegrated,) the elements of which, by some further chemico-vital process, resolve partially into oil, or fat globules." P. 41.

Mr. Gulliver says :—

"In the human subject, it appears to me that crude tubercular matter, from whatever organ obtained, differs as little in its microscopical as in its general and chemical characters. When examined by the aid of the microscope, crude tubercular matter can scarcely be said to present any regular structure, as it is merely made up of minutely granular matter, oily spherules, some shapeless albuminous flakes, or shreds, and a few irregular corpuscles; the latter are probably nothing but effete, or shrunken primary cells." P. 41.

As to the Chemical Characters of scrofulous matter, we need say very little. Prout regards it as albumen, imperfectly developed; Gendrin as a mass of albumen, with an excess of salts. Bredon considers it to be an albuminate of potash or soda. Boiling water and acids coagulate it, when it has become somewhat softened.

Before passing on to other matters, we may remark that Mr. Phillips is of opinion that the deposition of the peculiar scrofulous product is always preceded by a sub-inflammatory or hypertrophic condition of the affected glands; and he maintains that, when this deposit has once taken place, it is never removed by the process of absorption; suppuration and ulceration are believed to be necessary for its ejectment.

*Is the Blood altered in Scrofula? and, if so, in what manner?* This very important question is discussed in the fifth chapter. Baumes has stated that the constituents of the blood are less intimately mixed in scrofulous than in healthy persons. Dubois, besides alluding to the diffluence and imperfect coagulability of the blood in the former, has also pointed out a change in the form of the globules: he found them "lenticular, the central depression, or spot, being extended beyond its natural limits, and so defined that they looked like wheels;" "many of them being also deformed, notched, or otherwise irregular."

Let us now hear the results of Mr. Phillips' own observations on this subject:—

"I have examined," says he, "the blood in sixty-seven instances of Scrofula, and although I have almost always observed a considerable deviation from the condition of healthy blood, the changes have not presented sufficient uniformity to induce me to regard any particular condition as specially characteristic of Scrofula; the changes are such as seem to belong to a tolerable extensive group of affections, all, it is true, being connected with disordered nutrition and debility."

"In almost every case, the coagulum was relatively small, the serous menstruum, large, the clot was usually very soft, almost diffluent; in a few instances only it was tolerably firm. In almost all cases, the proportion of globules was considerably under the healthy standard. The fibrin had not usually undergone much change; in a few cases it exceeded, in many more it was below the healthy standard. In most instances there was a considerable increase in the proportion of albumen; in almost every instance the proportion of salts was found to exceed the healthy standard; in some instances it was nearly doubled. At one time I thought I had clearly made out, that the proportion of chloride of sodium was deficient, and certainly in many cases it was so; but in many other cases that deficiency was not evident. If succeeding observations should establish a frequent co-existence, it might be well to carry out Russell's salt-water plan in the treatment of the disease; he conceived it to be efficacious. It may be, that salt water so used acts as a stimulant of the whole economy, increasing plastic energy and thus improving the vigour of the system." P. 57.

The upshot of the whole is, however, anything but satisfactory; for our author frankly confesses that he is not "in a condition to point out any particular state of the blood which is certainly characteristic of Scrofula;" the very same deviations from the composition of healthy blood, that are usually discoverable in that of Scrofulous patients, being present in anæmia and other diseases of debility, where no scrofulous taint can fairly be alleged to exist.

After such an admission, is it not indulging a little too much in mere speculation to assume "that the blood is changed before the deposit is made, that the accumulation of certain morbid materials in the blood constitutes what is known as the scrofulous diathesis or constitution, and that their deposition in the subcutaneous lymphatic glands constitutes what we know as Scrofula"?

Chapter VI. is devoted to the consideration of the question, "*Are pulmonary tubercle or phthisis and scrofula in their nature identical?*" Here we may first ask, why should the comparison be instituted between *pulmonary* tubercle only, and not rather between the formation of tuberculous matter generally, on the one hand, and scrofula on the other?* The only answer we can find is given in these words: "I exclude, therefore, from the present inquiry, disease of the mesenteric, the bronchial, and other glands existing in the thoracic or abdominal cavities, because I wish to consider Phthisis and Scrofula under their most decided characters." Mr. Phillips admits that no difference can be pointed out between the morbid deposit, in its early stage, in the mesenteric and bronchial glands, and that of genuine tuberculous matter in the substance of the lungs; and, although

---

* At the very close of the preceding Chapter, our author appears to admit the identity of scrofulous and tuberculous disease in general; for it is said: "it is certainly difficult to understand how the necessary, abnormal action, that causes the (*scrofulous*) deposit, can be developed in so many regions of the body, and often in so many different tissues, almost at the same moment of time, as we find in *tuberculous* diseases, unless something more than a local action was at work to determine the deposit." It is curious also, that in the opening paragraph of the following Chapter, the term Scrofula is expressly "extended to all those affections in which a *tuberculous* product is deposited in any of the tissues of the body."

he says that he has very rarely known scrofulous deposit in the subcutaneous glands exhibit the "grey smooth, translucent appearance," so characteristic of pulmonary tubercles when first deposited, we might almost appeal to his own definition of scrofulous matter, (as given at the beginning of his work,) as sufficient evidence of their close relationship, if not their absolute identity.

Some of the modern continental pathologists have endeavoured to point out certain differences between scrofulous and tubercular diseases; with what success, the following extracts will shew.

Lebert (whose work on Pathological Physiology we reviewed in our No. for January of the present year), says:—

"Diseases which are commonly regarded as scrofulous, may be ranged under three categories: chronic inflammations, in which nothing specific can be detected, and which are not really scrofulous; scrofulous diseases, properly so called; tubercular diseases, which though having a great analogy with Scrofula, ought to be separated from it as well for clinical, as pathological reasons. I believe scrofulous and tubercular diseases have a certain connexion, that they depend on a dyscracic state of the blood, which is, in both cases, either identical, or, at least, analogous. I regard scrofulous diseases, neither as simple chronic inflammation, nor as a form of tubercular disease; and I believe in the essentiality of Scrofula. Tubercular diseases, on the contrary, appear in many circumstances to constitute rather a form of scrofulous disease." P. 64.

Is this distinction very clear or intelligible? Professor Albers of Bonn has addressed a letter to our author on the question of the "identity or non-identity of Scrofula and Consumption." From this, we extract the following passage:—

"The most essential results of my investigation are these—that Scrofula and Tubercles exhibit, in their physiological as well as their anatomical properties, several differences, so that their identity, so absolutely adopted in modern writings, is not to be justified. As you merely desire to know the anatomical differences, I pass over the physiological ones.

"1. Scrofulous swellings, particularly those occurring in the mesentery and the diaphragm, are so closely connected with the lymphatic vessels, that quicksilver may be driven through them and the lymphatic vessels. Real tubercles, even when they are softened, never permit the passage of the quicksilver.

"2. Scrofulous swellings have always around them, as well as in their parenchyma, blood-vessels, which pass from the cellular texture, directly into them. The tubercle very frequently forms around itself a caul of vessels, often in a fibrinous layer, which separates it from the lung-parenchyma. Blood-vessels pass but rarely into a tubercle.

"3. The tubercle presents, under the microscope, separate minute tubes, which under a linear power of 550 times, prove to be cells. This is not the case in the scrofulous matter." P. 312.

Now with respect to any microscopical differences, surely it would be injudicious to attach very much importance to them; for each successive observer seems to have little else to do, than merely contradict the statements of his predecessor on this as well as on several other points in the history of the minute structure of pathological formations. Mr. Gulliver expressly says that "crude tubercular matter, from whatever organ obtained, differs as little in its microscopical as in its general and chemical characters. The drawing shews how nearly the microscopic elements composing crude tubercle of the lungs and of the lymphatic glands agree."

Professor Albers pushes his peculiar views considerably further than any other writer, as far as we know; for we find him asserting that, "an important difference between Scrofula and Tubercles is presented with respect to treatment. Scrofula proves curable in all forms, Tubercles almost in none; Scrofula lasts a long time, Tubercles hasten much quicker to an issue. Scrofulous swellings decrease considerably after cure, and often disappear seemingly; Tubercles, when stopped in their development, do decrease and ossify, but the decrease of volume is never so considerable as with Scrofula." It is quite needless to comment upon these assertions.

Our author, who evidently leans to the same side of the question as Professor Albers and M. Lebert, appears to rest his opinion, as to the difference between Scrofula and Phthisis, mainly upon certain pathological and statistical data, the truth of which is, to say the least, very problematical. He maintains that inflammation or hyperæmic vascularity of the parts affected invariably precedes the development of the one, and not that of the other.

"The many opportunities I have had for examining lymphatic glands, before and after scrofulous matter has been deposited in them, have satisfied my mind that before the gland receives the deposit it undergoes considerable change; it becomes enlarged, its vascularity is much increased, and its consistency is almost flesh-like; this change in its condition I conceive to be the result of inflammation. In this respect, then, there is a marked difference between scrofulous deposits in glands, and tuberculous deposits in the lung. I am aware that considerable difference of opinion has existed relative to the state of the lung at the point where tuberculous matter is deposited; yet few persons will be prepared to maintain that the deposit is commonly preceded by Pneumonia. The older authors seem to have thought the presence of inflammation necessary for the development of all accidental productions, and this idea was supported by Broussais; few, however, of the more accurate observers of the present day are inclined to doubt but that tuberculous matter is usually deposited in the lung unaccompanied by any sign of inflammation, or even active hyperemia. We have clearly no right, then, to assume the existence of antecedent inflammation, when we find the pulmonary tissue around a tubercle apparently quite healthy." P. 67.

With respect to the statistical data, to which Mr. Phillips evidently attaches considerable importance as evidencing a marked difference between Scrofula and tubercular Phthisis, it would be easy to point out some fallacies, which must throw discredit on conclusions derived from this source. We are told that the average number of deaths annually from phthisis, in England and Wales, may be set down at 59,500; while that from scrofula does not exceed 1200. Now, without alluding to the frequent existence of indubitable scrofula in consumptive patients, who for one moment will believe that such a statement as this can be thoroughly correct? Why, very nearly the number last-mentioned die, in the course of a twelvemonth, from Tabes mesenterica alone; not to mention other scrofulous diseases of the viscera, joints, and so forth. In short, the more that we see of medical reasonings built upon the Registration Reports—as hitherto made up—the less confidence are we inclined to place in their truth. This we say, not only in disparagement of the system, but of the imperfect and inaccurate returns that have frequently been received. So completely however has our author trusted to statistical calculations, that he has actually been led to believe, that "where there is a large mortality from consumption, there is a small mortality from scrofula, and *vice versâ*. To bear him out in this somewhat unexpected conclusion, he adduces, among other circumstances, the testimony of Mr. R. Martin to prove that phthisis is much less frequent in India than in our own country, while scrofulous disease is alleged, on the authority of another East India practitioner, Dr. Stewart, to be very frequent there. As we shall revert to the opinions of these gentlemen, when we come to allude to the influence of Climate on Scrofula, we shall not say more at present than that they are strikingly opposed to each other on one very material point.

If we required any other argument to prove the intimate connexion and relationship of scrofulous disease and tubercular phthisis, we should point to Dr. Baly's letter addressed to Mr. Phillips, in reply to certain interrogatories on the subject. The entire letter, indeed, proceeds on the assumption of this very point; and the numerous and very interesting facts, which the writer brings forward from his experience during the last five years at the Millbank Penitentiary, abundantly shew that the very same circumstances and conditions tend either to check or to promote the development and progress of Consumption and of Scrofula—the two diseases uniformly increasing or diminishing *pari* (or, at all events, *simili*) *passu*.

"The frequency of Scrofula and internal tubercular disease amongst the prisoners in the Millbank Penitentiary, was one of the first and most important facts which offered themselves to my observation when I commenced my attendance at the Institution in the spring of 1840. I found that the prevalence of the disease had already engaged the attention of the Medical Officers, and that a considerable number of prisoners who were most severely affected with it, had been separated from the rest, and placed in a distinct Ward, and that the system of discipline as it regarded them, had by medical suggestion been much relaxed. The experience of the four subsequent years, and the examination of the Medical Records of the Penitentiary, only strengthened the impression which I had at first received.

"The great amount of tubercular disease engendered by imprisonment in the Penitentiary is proved by the following facts. During eighteen years, 205 deaths occurred amongst the prisoners in that Establishment. Of this number, however, 31 deaths arose from the Asiatic Cholera, which was epidemic during the years 1832-4, and only 174 from ordinary causes. Now of the 174 deaths, 75 were caused by Consumption, and eight by other forms of tubercular disease. Again, during the same period of eighteen years, 355 prisoners were pardoned on the ground of illness, and of these, 90 laboured under Consumption, and 78 under external Scrofula. So that very nearly half the deaths, and the same proportion of the pardons on medical grounds, were due to external or internal Scrofula, or tubercular disease." P. 362.

Mr. Phillips lays a good deal of stress, in the working out of his position that Scrofula and Phthisis are in many respects different forms of morbid action, on the circumstance that the subcutaneous lymphatic glands are diseased in only a very small proportion of those who die from phthisis. But what does this prove?—nothing more, as far as we can perceive, than that when, from a certain cachectic state of the system, one organ is more particularly the seat of disease, another is generally less seriously affected with it at the same time. Dr. Renton of Madeira puts the question thus :—" may not your views of Phthisis being rare, when enlarged glands are common, arise from the same identical constitution wreaking its vengeance on the more external parts." Although few of our readers, we should suppose, will be inclined to adopt Mr. Phillips's views, it may be but fair to him, and not uninteresting to them, to present, in his own words, the following summary of his views :

" I apprehend," says he, " it has now been shown, by abundant evidence, that with the exception of the deposit itself, which, whether found in the lungs, or in a cervical gland, whether examined by the naked eye, by the microscope, or by chemical analysis, is very similar, the circumstances attendant upon the development of Scrofula and Phthisis are widely different. In Scrofula the gland undergoes considerable change, inflammatory in its nature, before the matter is deposited in it; in the lung we commonly find the tissue around a recent simple tubercular deposit unchanged by inflammation. We find, further, that in districts where the causes of Phthisis act with most intensity, those of Scrofula fall lightest; that the age when the ravages of Scrofula are most keenly felt is precisely that when the visitation of Phthisis is least to be apprehended; that the sex which suffers most severely from one of those diseases is least affected by the other. And beyond all this, there is the fact that, among the numerous victims of Phthisis, at least eighteen out of every twenty exhibit no marks of having suffered from Scrofula. It seems to me, therefore, that these facts constitute so clearly marked a difference between the two affections, that it will be most convenient, most conducive to scientific correctness, to consider them as affections possessing a certain general similarity of character, but no identity. It may be that they belong to the same family, so do Pleurisy and Pneumonia; but every one deems it desirable to make as clear a demarcation as possible between those diseases. I say the same of tubercular disease generally and Scrofula, between which the points of resemblance are strong, in so far as concerns the deposit; but in all else they are weak." P. 78.

Despite this attempt at distinction, Mr. Phillips himself, in many subsequent parts of his volume, seems to regard Scrofula and Phthisis as members of the same family ; for, at page 203, he actually uses the former as the *generic* term, and includes the latter, and Tabes Mesenterica, as different species.*

In Chapter VII., Mr. Phillips endeavours to shew that Scrofula is by no means so common or prevalent a disease in England, as in most other countiies of the world. After giving a variety of statistical particulars, he says :

" It is thus seen, that though derived from so many and such different sources, there is a striking concurrence in the results of the evidence I have collected, and that agreement constitutes a strong reason for believing that my data do, very nearly, represent the actual prevalence of the disease. We see that the Returns

---

* It may be worthy of notice that M. Lugol, in his lately published volume, —of which we gave a copious review in the Number of this Journal for January, 1845—has devoted a Chapter expressly to prove the identity of Scrofulous and Tubercular diseases. The same opinion is held by MM. Rilliet and Barthez, whose admirable work on the Diseases of Children we still more recently brought under our readers' notice. Let us, however, not forget to mention that the subject named, last year, by the French Royal Academy of Medicine for the Portal prize was, " The Analogies and the Differences between Tubercles and Scrofula."

of cases of Scrofula, found among our ordinary population, are singularly confirmed, not only by the Returns of Hospitals and Dispensaries, but also by the examination of Recruits and Convicts; and I think we are thus justified in regarding as near the truth our estimate of the prevalence of Scrofula, such as we have defined the disease. That is to say, that scars are apparent in about 1½ per cent.; that the subcutaneous glands are enlarged, so as to be perceptible on simple inspection in less than 3 per cent.; and that the glands may be detected by the finger in 24½ per cent. of those of the children of the poor who are under sixteen, and in 8 per cent. of those above; or taking the whole population, in 10 per cent.; and that something less than 3 per cent. of the people are under treatment for the disease in its various forms." P. 84.

He then adduces numerous details respecting the prevalence of the disease in other countries of Europe and of America, and alludes particularly to the numbers of recruits in the French army that are yearly rejected for having marks of Scrofula. He closes his remarks with these encouraging (would that they were *proved* to be true) words:

"It appears, then, that comparing Paris with London, the deaths from Scrofula, when compared with the population, are six times as many in the former as in the latter capital; and that for the whole of France, the marks of Scrofula presented by recruits are twice as many as among our own recruiting population.

"Is it not, then, abundantly proved, that the notion that Scrofula is eminently an English disease, is incorrect; and am I not warranted in stating that there is no country, so far at least as our information extends, in which the people are more free from the disease than in England and Wales?" P. 91.

*Is Scrofula on the increase in this country?* Without adducing the data which our author has collected together on this point, we shall merely give the conclusion to which he has arrived:

"Tried, then, by such tests as I have been enabled to apply, which though not strictly accurate, are the best we possess, and which, when used with caution, constitute a fair body of evidence on the point, the conclusion seems a fair one that Scrofula is much less prevalent in the present day than it was in the seventeenth and eighteenth centuries." P. 98.

The subject of the Etiology of Scrofula is treated at great length in Chapter IX. Nearly 150 pages are devoted to its consideration. With respect to the hereditariness or transmissibility of the disease from parent to child, Mr. Phillips, after exposing the extravagant absurdity of many of M. Lugol's hobby-horsical views on this subject, as we had previously done in our review of his work, propounds some peculiar notions of his own. He will not, for example, admit that "a scrofulous mother does *ordinarily* produce a scrofulous child."

"Nay," he says, "it is not apparent that the influence of a scrofulous parent is more efficient to induce Scrofula in the child, than the influence of equal constitutional debility in a parent, originating in other causes than Scrofula. In other words, an ailing parent is less likely than a healthy one to give birth to a vigorous child, and a weakly child is more susceptible than a vigorous one of Scrofula, as well as of other diseases. But in the sense of a direct specific tendency in a scrofulous parent to reproduce in the child the same disease from which the parent suffers, in virtue of an agency of a different nature to that which would be exercised by simple constitutional debility in the parent, it seems to me we have no satisfactory proof." P. 238.

According to his researches, the influence of hereditary transmission does not appear to be quite 4 per cent.—a very low ratio, it will be admitted. As bearing him out in his views, he appeals to the testimony of M. Louis in reference to tubercular phthisis; for this gentleman remarks that "in reality I have observed nothing decisive in favour of the hereditary character of phthisis." After examining the opinions of various other writers on the general question of hereditary influence and predisposition,* Mr. P. comes to this conclusion respecting Scrofula:

"The result of the facts now offered is, that a feeble constitution is a favourable, though not a necessary, or indispensable, condition for the development of the tuberculous cachexia; that a feeble constitution is more likely to be the offspring of diseased than healthy parents; but that we are not in a condition to point out the causes in the parent which tend most to entail feebleness upon the child, nor what kind of disease or feebleness in the parent tends most to induce the development of Scrofula in the child." P. 127.

We need not extend our remarks on the various alleged causes of scrofulous predisposition in the child, as derived from the age or affinity of the parents, from being suckled by a scrofulous nurse, and so forth,† as our attention was so recently occupied with all these matters in the review of Lugol's work; and there are no new facts adduced by the present author to throw light upon these somewhat obscure topics. There is, however, one opinion that may deserve to be noticed *en passant*, as it is decidedly at variance with common belief.

"I doubt whether we have any clear evidence of the bad consequences, either on the mind or the body, of frequent intermarriages. I know that, in the management of stock, a notion adverse to breeding in and in prevails, but I know also that it is most extensively practised, especially with the best bloods and breeds, and therefore I conclude that the apprehended evils are not realised.

"In so far as concerns the human race, this point is not easily elucidated, and even with reference to brute animals the evidence is very inconclusive. The only mode of satisfactorily examining the question in the human species, is to observe people who are more or less isolated, and are therefore obliged to intermarry within a narrow circle. For if the principle be operative, it should be found under these circumstanees. The classes most favourably situated for the inquiry are the inhabitants of small islands, having little intercourse with other islands and main lands; and on our own coasts, the people of the Isles of Portland, Man, and of the Channel Islands best realize those conditions, but I do not find that Scrofula is more prevalent among those Islanders, than in places where the

---

* The following truly characteristic and instructive anecdote of John Hunter well deserves to be generally known:

"On the trial of Donellan for the murder of Sir Theodosius Boughton, he was asked: 'Is not apoplexy sometimes apt to run in a family?' To which he replied: 'There is no disease whatever that becomes constitutional, but what can be given to a child; there is no disease that is acquired, and becomes constitutional in the father but can be given to a child. The father has a power of giving that to the child by which means it becomes hereditary. There is no such thing as hereditary diseases, but such a thing as hereditary disposition." P. 122.

† "Dupuy asserts that 'the milk of a tuberculous cow may contain a greater quantity of phosphate of lime than that of a healthy cow. Labillardière has proved that there may be seven times more phosphate of lime in the milk of a cow affected with pulmonary tubercle than in that of a healthy animal." P. 141.

What should cause this inordinate amount of the earth of bones in the milk of a diseased animal?

circle is much larger. The Quakers, again, have for years married within a comparatively narrow circle, certainly much narrower than other classes of people in Britain; and I do not find more Scrofula among them than among other people of a similar social condition. The Jews, again, marry within a narrow circle, and in their early history near intermarriages seemed to be sought for; and we have no authority for saying that Scrofula is more than usually prevalent among that isolated race. And with respect to noble and royal families, I know of no evidence which favours the belief that frequent intermarriages tend to the production of Scrofula." P. 138.

From this debatable point, we pass on to notice some of the remarks of our author on the influence of *Alimentation* in the production of Scrofula.

It is almost unnecessary to say that those infants, who are deprived of their mother's milk and are brought up by hand, not only have a much more slender chance of life than those that are suckled as Nature intended, but also, if they should survive, are generally more feeble, ailing, and more frequently affected with Scrofulous maladies. No one is likely to dispute either the fact—for it has been proved over and over again—"that Scrofula is least prevalent where children and others are best fed," or the position "that food exercises a more important influence than any other agent in the production of the disease." In reference to these points, Dr. Baly mentions a very conclusive fact, derived from his experience of the prisoners in the Millbank Penitentiary, which in itself speaks volumes: "A marked difference in respect of their general health and the number of them affected with scrofulous disease, is presented by the convicts sent to the central prison of Millbank, from different parts of Great Britain, preparatory to their transportation. By far the thinnest convicts, *and those having the largest proportion of unhealthy and scrofulous individuals* amongst their number, come from the Scotch prisons, in which the diet consists of a sparing allowance of vegetable and farinaceous food."

As shewing the paramount influence of diet on the production of scrofula, we have only to attend to the state of things among the poorer classes in Ireland. The disease is more than twice as common among the Irish as it is among the same classes in England; we need not say that the food of the former is vastly less nutritious. The following communication from Dr. Martin of Waterford to our author, attests the distressing amount of scrofulous disease among the children in the Portlaw district.

"In 100 children under thirteen years of age, of people of the poorest class, not employed in factory labour, 17 had fair hair and blue eyes; enlarged glands were to be felt in the necks of 83, and 2 had suffered from necrosis of the tibia. In 50 children under 13, in the class of small farmers and petty shopkeepers, 28 were of fair complexion and grey eyes; enlarged glands were detected in 18 of the 50; 1 suffered from morbus coxæ. In 60 children, under 13, belonging to the parents of the poorest class of factory operatives, 8 had decidedly fair complexion and blue eyes, the majority had light brown hair and grey eyes; enlarged glands were to be detected in the necks of 37; 1 had a scrofulous cicatrix, and 1 was suffering from white-swelling. In 60 children, under thirteen years, of the more comfortable class of factory operatives, 8 had decidedly fair complexion and blue eyes; enlarged glands were to be detected in the necks of 26. In 100 girls, between fourteen and eighteen, employed for more than a year at factory labour, 9 were of decidedly fair complexion and blue eyes; enlarged glands were to be detected in the necks of 79, scrofulous cicatrices in the necks of 2; marks of necrosis in 4, namely, in 2 instances affecting the femur, in 1 the tibia, and 1 the metacarpal bone. In 100 boys of the same ages, 14 were of decidedly fair complexion and blue eyes; enlarged glands were to be detected in the necks of 71; none had cicatrices in the neck; 3 had necrosis of the tibia, 1 of the humerus. Altogether 470 children under eighteen years of age; of whom 315, or 67 per cent., had marks of Scrofula—14 having scars." P. 178.

Mr. Phillips appears to attach much less importance to the influence of *impure air* from crowding, imperfect ventilation and so forth, in favouring the production of scrofulous disease than most writers. According to his enquiries, "neither the general mortality, nor the deaths from scrofulous diseases, bear any definite relation to the closeness with which the population is crowded together, whether the comparison is made between one Town or District and another, or between different portions of the same Town or District."

The remarks of M. Carmichael, however, subsequently quoted by our author, very forcibly illustrate the paramount importance of exercise in the open air in a hygienic point of view:

"From some observations I have made on other Institutions, for instance, St. Thomas's Parochial School, and the Bethesda School, to which I was medical attendant, I came to the conclusion that depriving children of that active exercise in the open air, which is so necessary to their health and development, is almost as injurious as improper nutriment. Let a healthy child have sufficient exercise, and his powers of digestion are so sharp, that he will perhaps assimilate the most inappropriate diet: otherwise the majority of the children of our poor would become scrofulous: deprive him of his liberty, and his nutriment will remain undigested, and occasion all the symptoms I have mentioned. The children of both schools were fed, clothed, and taken the best possible care of, with this exception, that from the want of play-grounds, they were prevented from the enjoyment of active exercise; and although free from disease at the time of admission, near one-third of their number was found exhibiting the symptoms of Scrofula. They were marched out, no doubt, when the weather permitted, once a day, in a sober funeral-like procession; but let no person imagine that such dismal, boarding-school exhibitions, are sufficient for the health of children." P. 253.

The section on the Influence of *Occupation* upon the production of Scrofula contains some very curious details, the results of which will surprise most readers. That the sort of employment and the mode of life generally have much to do with the mortality of a district, independently of its local salubrity or otherwise, is abundantly proved by the well-ascertained fact that the value or expectation of life, at the age, say, of thirty years, varies a good deal in different classes of the community: it is *least* among the nobility, and *highest* among the provident, regularly-employed, artisans of our towns, and agricultural labourers! "Among the latter it is 40·6 years; among the members of Friendly Societies, including all trades, it is 36·6; among professional men it is 33·9; among the gentry it is 31·2; whilst among the Peerage it is only 30·9. The average for England and Wales being 34·1, Scotland 33·1, Ireland 31·7; but the expectation of life at thirty in the poor fork-grinder of Sheffield is only seven years. Thus a town of fork-grinders, however well it may be built and drained, and however well the people may be fed and lodged, if judged of by the mortality, would be pronounced a very unhealthy district. So those districts of our country in which the bulk of the population are miners, might in the same way be regarded as insalubrious, although the real cause of the excessive mortality was mining, which is unfavourable to longevity. Again: there are districts where the population is mainly composed of agricultural labourers, of all occupations the healthiest, and if we suppose two hamlets, in the same district, the one the abode of miners, the other of agricultural labourers,—although in all else than the occupation of the people, these hamlets are similar, yet, at the age of thirty, the expectation of life will differ to the extent of seven years, and this although the miner will be better fed and clothed than the agricultural labourer."*

Although, however, the value of life is considerably higher in rural districts than in large towns, it would seem, from our author's investigations, that Scrofula is decidedly more common among our agricultural than among our manufacturing population. This may arise from the circumstance of the food of the former being less animalised, and of a poorer description altogether, than that of the latter.† The greater amount of mortality among the inhabitants of towns is due not to Scrofula, but to other diseases, induced by dissipation, intemperance, residence in a vitiated atmosphere, &c.

It is a very general belief that employment in large Factories tends strongly to develop scrofulous disease, and thus to shorten life very materially. Upon enquiry, it would seem that there has been a good deal taken for granted in arguing upon this head. Mr. Phillips has collected a

---

* Dr. Guy remarks that, in the Peerage,

| | | |
|---|---|---|
| " At 20 the expectation of life is | 38.47 years. | |
| 30 | ,, | 30.87 ,, |
| 40 | ,, | 24.45 ,, |
| 50 | ,, | 17.92 ,, |
| 60 | ,, | 12.56 ,, |

While, at corresponding ages, among the general population, a very large proportion of whom are deprived of advantages enjoyed by their more elevated countrymen,

| | | |
|---|---|---|
| At 20 the expectation of life is | 40.69 years. | |
| 30 | ,, | 34.09 ,, |
| 40 | ,, | 27.47 ,, |
| 50 | ,, | 20.84 ,, |
| 60 | ,, | 14.58 ,, " |

According to the experience of Friendly Societies, the expectation of life in rural districts, at 30 is 38.4 years; and in cities 32.8 years.

† "The dietaries of Union Workhouses, as contained in the Poor Law Circular, having been compared with the food of the agricultural labourer, and the prevalence of Scrofula in each condition having been shown, we arrive at this result, namely, that the Workhouse child is better fed, and less subject to Scrofula than the child reared in the cottage of the peasant: and when it is considered that many pauper children are received into the Workhouse when suffering and destitution have probably developed disease, and have almost certainly produced debility, the comparison is even less favourable for the child of the independent labourer, than the result which is shown by the numbers actually examined."

great deal of very interesting information, that throws considerable light upon the subject. After alluding to the cases of Manchester and Liverpool—the one a factory town the other not—where the mortality is found to be highest in the latter, he continues: "Take again Bristol and Leeds, the one with a Factory population, and the other with a population not engaged in Factory Labour, and we find the mortality in the former 2·91, in the latter 2·59 per cent.; while at Bath, famed for its salubrity, with no Factory, and with a smaller juvenile population than Leeds, the mortality is 2·35 per cent. Still it is certain that the mortality of large Factory Towns is generally large; but it must be born in mind, that it is not uniformly larger than that of similar towns in which Factories do not exist. If we take an equal number of Factory and Non-factory Towns—each group including a population of upwards of two millions, we find that the relative mortality is as 2·5 to 2·4; and this although the influence upon the general mortality of a rapidly increasing population is usually much greater in Factory Towns than in others, because the proportion of children to adults is greater in a rapidly increasing population, than in one where the population is stationary, or where its progress is less rapid."

The result of all his tabular statistical comparisons is, that "if there be anything peculiarly injurious to life in Factory towns or Factory labour, it is not then made apparent by a register of deaths. The injurious effects of Factory Labour, when compared with other laborious occupations, are not apparent in our Mortality Tables, or in the Returns of Friendly Societies. And the mortality in Factory Towns appears to press just as heavily on masons, shoemakers and tailors, in those towns, as upon those persons who are employed in Factories, and it would therefore seem improper to refer to manufactures an evil, which is not peculiar to them."

With respect to the question whether Factory labour tends to induce or promote the development of Scrofula, Mr. Phillips frankly admits that it would not be difficult to establish very opposite results from the evidence taken before different Factory Commissions. So much, we may again remark *en passant*, for the reliance to be placed upon many of the results of statistical enquiries, as hitherto conducted. In France, MM. Baudelocque and Villermé regard Factories as, in general, seminaries of scrofulous disease; whereas M. Lugol tells us that Scrofula is by no means common among the workmen employed in these establishments, even when they are, at the same time, badly fed, housed, and clothed. "In fact," says this gentleman, many of whose assertions are to be received *cum grano salis*, "in my own practice, I see most of it (Scrofula) where the comforts of life are possessed." But M. Lugol is not singular in his opinion as to the effects of factory labour upon health. Dr. Carbutt, physician to the Royal Infirmary at Manchester, addressed the Factory Commissioners, some years ago, to the following effect:—

"The fact is, that Scrofula is almost unknown in Cotton Factories, although the climate of this town and neighbourhood is particularly cold and humid. In a very extensive examination, which I and some other medical men made a few years ago, we found, to our surprise, that the Cotton Factories, instead of producing Scrofula, are in some sort, a kind of means of cure. The late Mr. Gavin Hamilton, who was for thirty-six years Surgeon of our Infirmary, and who, previously to that, had been Surgeon in the Queen's Bays, said in my hearing, after examining the Cotton Factory, 'Gad we found the Cotton Factories to be a specific for Scrofula.'" P. 229.

The remarkable absence of scrofulous disease among those employed in factory labour, Dr. C. attributes "to the dryness and warmth of the Cotton Factories, to the lightness of the work, and to the superior food and clothing which the superior wages of the work-people enable them to obtain." The testimony of several other professional men is brought forward, in confirmation of the same views. For example, Mr. Poyser remarks of the workpeople in Mr. Arkwright's mills:—

"The result (of the examination) was extremely satisfactory to me, and confirmed me in the opinion I have maintained and before expressed to you, that the employment in these Factories, so far from producing or aggravating Scrofula, has a tendency to prevent it. The light, dry, and airy rooms in which the hands are employed, the constant exercise they take in moving from one spindle, &c., to another, and the good food and clothing their wages enable them to procure, are I apprehend among the causes which induce this immunity from Scrofula." P. 360.

That all medical visitors of Factories have not been able to report so favourably as these gentlemen, will not surprise any one. Mr. Phillips acknowledges that "the evidence collected by the gentlemen who were employed on the 'Children's Employment Commission,' is less uniformly favourable; and although the preponderance is in favour of the better relative condition of Factory Operatives, when compared with other classes of the labouring poor, yet that evidence is of so contradictory a character, that it might be plausibly used in support of either view of the subject." It is on the whole, however, pleasing to find, from the testimony alike of French and English writers, that the establishment of large Factories has, on the whole, tended to improve, not to deteriorate, the physical condition of the working classes. That much might be done to make that condition better in respect alike of mind and body, will be denied by no one; but let us not meanwhile be led into error on the general question, from confining our attention solely to the many evils that may still exist. M. Villermé's remarks on the past and present condition of the factory labourer in France apply, with equal force, to our own country.

"Whenever large numbers of people are collected into narrow spaces, unless there be any counteracting influence in operation, their health suffers. If we would extend this assertion to the manufactories, the facts which have been made known are far from always confirming it. There is, perhaps, no disease which belongs to a particular factory, but there are diseases which are more frequent, when the conditions of life of the labourer favour their development. But almost every disease will prevail in a crowded Non-factory Town, in an equal degree with a crowded Factory Town; and even in Factory Districts, it is upon those who are not employed in Factories, that the mortality falls with most severity. And this seeming incongruity is easily explained; those who are not employed in Factories, are equally exposed with those who are, to noxious local influences; but the earnings of the Factory Labourer are such, as to give him the means to overcome many of the influences of noxious agents, under which the idle, or the irregularly employed, or very poor, would sink." P. 234.

"There are," he continues," many Factory Labourers whose gains are so small, that they are scarcely sufficient to procure strict necessaries. Are they, however, more wretched and proportionally more numerous at present than formerly? There is no proof that they are. It is well that the labourer should know that his present condition is better than it has ever been. The documents from whence we may deduce a knowledge of his condition at different periods furnish the proof. I have been struck at different places, which I have visited before, to see the workman eating better bread, wearing stockings where formerly one only saw naked feet, shoes where there used to be sabots, cleaner, lighter, better furnished rooms. I found, in fact, in all these places, not what I could have wished, but much less that was bad, than twenty or thirty years ago." P. 235.

So much for the influence of occupation in the production of Scrofula: let us now turn our attention to another topic, which of late years has excited much interest.

It affords a striking—and, we cannot but add, a *humbling*, as respects the value of medical evidence—example of the discrepancy of different writers on the exciting causes of Scrofula, if we compare what Lugol says of the influence of *Prison Confinement* with the experience of Dr. Baly, as recorded in the letter to which we have already referred. The great (as some folks imagine) continental authority says:—

"We know that houses of detention are generally very humid; that they unite, if not all, the greater number of the things which are regarded as occasional causes of Scrofula, misery in every form, privation of air, of light, of exercise, the influence of heat, cold, and of damp, of coarse food, and in too small quantity, dirty and insufficient clothing in winter, a bad bed to lie on, and the deepest demoralization. The union of these causes occasions many diseases in prisons; Scabies, Prurigo, Dysentery, Chronic Enteritis, Putrid and Jail Fevers; but to this list we must not add Scrofula: for not only is it not endemic in prisons, but we scarcely see a case there, and it is equally rare in the dampest and most unhealthy workshops." P. 237.

How different is the evidence of the Medical Superintendent of our Millbank Penitentiary, as we have already shewn by the extract from his letter, which we have given a few pages back!

"On examining the statistical Reports of the Penitentiaries of other countries, I have found that in them also the scrofulous or tubercular diseases have been the principal cause of death, and that the mortality from these diseases has been twice or three times as great among the prisoners in those situations, as amongst persons of the same period of life in the general population of the respective countries." 363.

As to the influence of *climate* on the production of Scrofula, the general impression hitherto among medical men has been that cold damp regions, exposed to frequent alternations of temperature, are, *par excellence*, its favourite abode, while such as are warm and dry are comparatively exempt from it. But now all this is alleged to be a mistake.

We have already seen what Mr. Phillips says respecting its *alleged* prevalence in our own country; he recurs to the subject in a subsequent part of his work:—

"England is always pointed at as an illustration of the first condition, whilst the plains of India have been regarded as a good example of the second; it being assumed that there is much Scrofula in England and but little in India. England and India may really exemplify those opposite conditions of the atmosphere or climate; but it has been already shown, that the prevalence of Scrofula is greatest in India, where it has been assumed to be least, and least in England, where it has been assumed to be greatest." P. 207.

A little farther on, he again vindicates the cause of his country:—

"It has been usual to point to Holland and to England, as cold and damp countries, in which Scrofula is more than usually prevalent; and the prevalence being assumed, a cause was also assumed, and that cause was humidity. But although we may admit that those countries are comparatively cold and damp, we deny the unusual prevalence of Scrofula, at least in England, because it is now demonstrated, that in no European country do the people suffer less from Scrofula than in England." P. 213.

Mr. Phillips admits, however, that cold weather favours the development of the disease; for we find it subsequently stated that "whoever considers the question fairly, will be struck by one fact, which cannot be denied, that, in European countries at least, scrofulous diseases are evolved or aggravated, during the cold of Winter."

As we mentioned before, there is no little discordancy among Mr. Phillips's correspondents as to the prevalence of Scrofula in the East Indies. Mr. Martin expressly states, as the result of very extensive observations, during many years, on the natives of Bengal, both civil and military, that "Scrofula, as an idiopathic disease, is seldom seen amongst them." He goes on to remark:—

"At the Native Hospital of Calcutta, of which I was Surgeon for ten years, I saw, however, many cases of scrofulous disease amongst the poorer Bengalees, caused, as it appeared to me, by the abuse of the rude preparations of Mercury and Arsenic, so liberally administered by the native empirics. Arsenic is given liberally in every form of fever, carefully avoiding evacuants, and as carefully excluding ventilation. It will be no matter of surprise then, that the survivors from such treatment should be troubled in after-life with various glandular enlargements. Of Mercury a rude sort of Chloride is prepared by the native doctors, containing a goodly proportion of Bichloride. This mineral is quite as freely exhibited as the first-mentioned, and with fully as evil an effect on public health amongst the indigent natives.

"In all rheumatic cases, in eruptive diseases, as well as in every chronic ailment that puzzles the empiric, this horrible preparation is given in large quantities, and often alternated with Arsenic, while salivation and enlargement of the glands, consequent on this treatment, are never considered as reasons prohibitory of bathing in rivers or tanks even during the cold season. It is needless to describe the lamentable consequences, or the frequency with which protracted suffering from glandular disease and premature death ensue.

"Now as to European residents in India; the civil and military inhabitants of the better classes are almost exempt from Scrofula, and so are their children. This exemption is equally true of parents and their offspring in Bengal, whose families in England are notorious sufferers. During an extensive observation of twenty years in the capital of British India, I do not remember three instances of scrofulous disease declaring itself, though numberless persons were known to me in whom the disease remained latent; and, as it appeared to me, solely through the influence of climate." P. 339.

He then compares the mortality arising from "every disease that can fairly be considered strumous"—viz. phthisis, hæmoptysis, scrofula, hydarthrus, and atrophia—among the British troops (European and Native) in the East Indies, with that among our soldiers at home; and what is the result of this comparison?

"The comparative exemption from pulmonary disease of those serving in Bengal in particular, is very remarkable; the ratio of admissions per 1000 being only 1·8 per annum, while in England, it is 6·4; and the annual deaths, which in England average 5·3 per 1000 by this disease, are all over India 'too small to calculate into Ratios.'

"The ratio of death by all descriptions of scrofulous disease in India generally is 1·6 per 1000 annually, whereas in England it amounts to 5·7." P. 340.

Mr. Martin concludes his letter to our author with these truly practical remarks, which, be it remembered, perfectly coincide with the opinions held by the profession generally.

"On the question of the general influence of the climate of India in scrofulous disease, I would observe in conclusion, that it is pre-eminently beneficial. The equable determination to the surface relieves from glandular obstruction and disease, while the phlegmatic of habit, with dyspepsia, languid circulation, and cold extremities, improve under a residence within the Tropics. The weak chested, as they are called in England, and such of them especially as are of scrofulous habit, are saved by going to India; and I have known instances without number in the curable stage of consumption, that is, labouring under the preceding stage of 'Tubercular Cachexy,' to enjoy good health in Bengal, and to survive their brothers and sisters at home. The fate of those, on the other hand, who go to India with suppurative Tubercles, or even in the stage immediately approaching to it, is only precipitated." P. 341.

We thus see that Mr. Martin is clearly of opinion that "scrofulous disease is *not* very frequent in Indias." Drs. Stewart and Spry have however come to a somewhat different conclusion. The former of these gentlemen observes :—

"In short, all half-castes in Bengal may be said to be scrofulous, though the disease does not develop itself so early in this climate as at home, in the forms you describe, but in cutaneous troublesome sores, weak eyes, mesenteric diseases, spleen, &c. What is very striking is, that 3 half-caste children will exhibit these symptoms for 1 English one, though all 4 be equally carefully brought up, fed, clothed, and tended.

"The climate seems to have a favourable effect in retarding, if not even arresting entirely the development of Scrofula in English children, while it has exactly an opposite effect on half-castes. I will not pretend to account for this, though it would be easy to theorise, but I am sure that I have seen the lives of several English scrofulous-looking children saved, by keeping them in Bengal instead of sending them home, and I have known many scrofulous-looking half-caste children turn out stout fellows who were sent home very young, and must have grown up to be consumptive striplings had they been kept in India." P. 343.

After giving some figures, he adds :—

"Thus it is evident that the scrofulous constitution is the prevailing one in Bengal, a fact well known to all Indian practitioners, and that among those who have comparatively soft skin, thin hair, and light brown eyes, the proportion is great." P. 343.

Dr. Spry examined 75 children of mixed parentage, of whom *all* had swelled cervical glands; 4 had open scrofulous sores; 136, of pure English parentage, of whom none were scrofulous; and 504 native children, of whom no fewer than 300 were scrofulous. Dr. Jackson's report is to a similar effect:

"I have examined, at different schools, indiscriminately, under ten years of age, 100 boys, all born in India, and of Creole extraction, of whom 80 may be called of dark complexion, eighteen have flaxen hair and olive eyes, 2 very fair, with grey eyes. Of the 80, a *majority* are subject to glandular disease; of the 18, none are free from glandular tumors, but not suppurating. The 2 fairest are also scrofulous."* P. 90.

We cannot reconcile these and such-like discrepancies, without supposing that different medical men are not agreed as to what diseases are to be considered as scrofulous, and what are not. It seems to us, therefore, that it would have been judicious on the part of our author, had he hesitated more in placing much reliance on some of his statistical positions. Can we, for example, after what we have just read, yield our assent implicitly to the assertion, that "in India, in China, in Russia, in Greece, hot, cold, and temperate countries, Scrofula is unusually prevalent? It is comparatively rare in Barbadoes with a warm climate, in New Brunswick with a cold, and in the Bermudas with a temperate climate."

Many other instances might be quoted where, by trusting too unreservedly to reports of insufficient authority and trustworthiness, Mr. P. has been led, we think, to adopt conclusions that will not stand the test of future scrutiny. Nevertheless, the highest praise is due to him for the great labour and earnest zeal that he has expended upon the object of his enquiries. The very difference of opinion elicited cannot fail to do good, by directing more general attention to the subject.

The concluding Chapter of the present treatise is occupied with the important subject of the *Treatment* of Scrofula. Our readers will not expect that we do more than merely glance at some of its contents, as we had occasion very recently, while examining the works of M. Lugol and Dr. Smith, to discuss the various remedies and hygienic means that are generally employed for the prevention and cure of this cachexy. Mr. Phillips is too sound a practitioner to have any novelty to propound, or any favourite remedy to recommend. In all his remarks, he displays much good sense and discriminating judgment.

He is far from thinking so highly of Iodine and its preparations, as most practitioners, we fear, do.

"What the exact influence of Iodine is in Scrofula, it is difficult to determine —I mean when not administered in combination with other substances than Potass. I am satisfied, however, that in many cases under the influence of Iodine, the tongue will become much cleaner, the appetite will improve, and the secretions will acquire a healthier character. And the impression left on my mind is, that the good which may be experienced from the use of this medicine, is not owing to any specific influence which it exerts over Scrofula, but to its occasional power of modifying the mucous surfaces, so as to enable them to assist in producing healthy nutrition.

"Whatever good may be derived from Iodine when uncombined, I think that, when associated with particular substances, with Iron, for instance, its power

---

* "I think 8 out of 10 half-cast children scrofulous.
5 out of 10 native kind.
4 out of 10 English.
1 out of 10 Mussulman."

over the disease may be increased; but it would be difficult to prove that there are not other forms of Iron which act as favourably as the Iodide in cases of Scrofula. If that impression be correct, as much, if not more, of the benefit may be owing to the Iron as to the Iodine." P. 276.

As to Iodine having direct anti-scrofulous virtues, we quite agree with Mr. Phillips in his negative conclusion.

The muriate of Barytes is regarded by him as little, if at all, inferior to Iodine, as a discutient of scrofulous glandular tumours. The muriate of Lime is believed to have very little, if any, efficacy. Alkalis are often useful, *if* their use be associated with a tonic regimen, and exercise in pure air; but then, how much of the benefit is to be attributed to the medicine? According to our author's experience, Alkalis have been mostly useful in cases "in which much acidity pervaded the secretions, and acted upon the general economy." Cod-liver oil unquestionably produces good effects in some cases: its *modus operandi* is then ascribed by Mr. P. to its power of improving the digestion and nutrition, and not to its having any direct or specific anti-scrofulous virtues from the Iodine which it contains, or otherwise.

With respect to the effects of residence at the sea-side, bathing in the sea and drinking sea-water, our author will not agree in the extravagant praises that have been bestowed upon them by some writers:—

"I am by no means convinced," says he, "that the sea-side is more desirable for the residence of persons suffering from Scrofula, than healthy inland situations. I have been accustomed to send scrofulous patients to the sea-side, because it is usually a thorough change of air, and on their return home, I have commonly found a certain improvement in their general health; but the glandular tumours, though reduced, were usually still present. I do not mean to say that, in the majority of the patients sent, the tumours still remained, but this has certainly been the case in a large minority; and even in those patients where the cure has been apparently most complete, the tumours have frequently re-appeared during the following winter or spring."

After alluding to various other remedies that have, on the whole, been far too highly praised, he sums up in these words:—

"If change of air, good food, and exercise, cannot be procured, the difficulty of treatment is in a ten-fold measure enhanced, and the chances of cure infinitely lessened. For good food, pure air, and proper exercise, the vaunted anti-scrofulous specifics are a poor compensation; we may try one after another, and often find all fail. All that is left to us in such cases, and unhappily they are many, is to endeavour to improve the mucous surfaces and the blood by alteratives, and tonics. In this way, we can do some good; but it can avail but little to labour by medicine to make the stomach fitter to digest good food, when the patient cannot procure such food." P. 305.

In closing our notice of this volume, we cannot but again express our very cordial admiration of the industry, talent, and zeal—philanthropic no less than professional—displayed by its author. We have not agreed with him upon several points, and we have frankly expressed our difference of opinion. This is indeed nothing more than what Mr. Phillips expected himself; for he candidly acknowledges that several of his positions might be found wanting in precise accuracy. We trust that Mr. Phillips may be encouraged, by the success of his present work, to apply his active mind to the elucidation of some other subject of medical enquiry.

